# The antioxidant system was unexpectedly strongly suppressed in *apis mellifera* worker bees emerged from larvae reared on combs adulterated with paraffin or stearin

**DOI:** 10.1038/s41598-025-08596-w

**Published:** 2025-07-01

**Authors:** Aneta Strachecka, Patrycja Staniszewska, Krzysztof Olszewski, Magdalena Chęć, Mariusz Gagoś, Piotr Dziechciarz, Maciej S. Bryś, Jerzy Paleolog

**Affiliations:** 1 Department of Invertebrate Ecophysiology and Experimental Biology, University of Life Sciences in Lublin, Doświadczalna 50A, Lublin, 20-280 Poland; 2 Subdepartment of Apidology, Institute of Biological Basis of Animal Production, Faculty of Animal Sciences and Bioeconomy, University of Life Sciences in Lublin, Lublin, 20-950 Poland; 3 Department of Cell Biology, Institute of Biological Sciences, Maria Curie- Skłodowska University, Akademicka 19, Lublin, 20-033 Poland

**Keywords:** Antioxidative enzymes, DNA methylation, Vitellogenin, Wax adulteration, Honeybee, Entomology, Enzymes, Biochemistry, Physiology, Biomarkers

## Abstract

The bee-wax combs are “the biggest organ of the bee colony body” as, in addition to their structural functions, they transfer information – pheromones and sounds. The lack of quality control procedures for bee-wax foundation, leads to a deterioration of this raw material, among others with the addition of paraffin and/or stearin. The adulteration of beeswax, from which wax foundation is produced, affects the mechanical strength of the combs and the development of the brood. Little is known about the effects of these adulterants on bees’ biochemistry and physiology. Therefore, the activity of the antioxidant system (SOD, CAT, GPx, GST and Vg) was determined in the hemolymph of bees reared on pure wax and wax adulterated with paraffin (10%, 30% or 50%) or stearin (10%, 30% or 50%). Additionally, the level of global DNA methylation in the brain of these bees was identified. We showed for the first time that paraffin or stearin strongly suppressed the activity of the antioxidant system, including Vg, in honeybee workers emerged from larvae reared on combs adulterated with these compounds. Stearin was much more harmful and may cause serious metabolic disturbances, including an increase in the global DNA methylation. This is important new information that serves as a warning to wax foundation producers and beekeepers. Therefore, there is an urgent need to introduce proper procedures and regulations for the routine quality evaluation of wax intended for the production of the bee comb building foundation.

## Introduction

The increasingly frequent depopulation problems among honeybee (*A. mellifera*) colonies stem from many reasons, which therefore were intensively studied^1,2^. Apart from diseases, harmful anthropogenic factors, such as pollutants, harmful xenobiotics, including particularly pesticides and heavy metals, but also plant monocultures and a reduction of honeybee food resources, were considered^3–6^. It must be noted that the bee-wax combs are believed to be the essential “biggest organ of the bee colony body”, as in addition to their structural functions, they transfer information – pheromones and sounds^7^. However, the question arises whether, as a result of human activities, wax could become one more harmful factor to the bee colony. The discussion of this problem is not sufficient, since it has focused on bee-wax as a raw product for beeping and other industries^8^but not on its impact on honeybee physiology in the context of its anthropogenic contaminations. Furthermore, the lack of procedures and proper regulations for routine quality control of the manufactured bee-wax substrates, lead to a deterioration of this raw material, mostly brought about by wax foundation producers^9^. Consequently, what is the most important for answering the above question is acquiring more information on the adverse effects of bee-wax contamination with admixtures of anthropogenic substances on honeybees’ health at every stage of their development^9,10^. Paraffin or stearin are such substances. They are often added to the wax foundation because they do not change its properties during the first superficial organoleptic assessment. The scarcity of bee-wax on the market stimulates this practice. The producers usually do not inform consumers about the application of such admixtures. Hence, they have been termed as bee-wax adulterations^10–12^. The addition of paraffin to the wax foundation, however, reduces the physicochemical properties and mechanical strength of the combs^13,14^. On the other hand, the impact of these wax adulterations strongly disrupts the brood development^9^. Chęć et al.^9^ showed that the addition of 10% of stearin to the wax foundation caused a 67% decline in brood survival. Furthermore, brood survival in combs built up on wax foundations containing 30% and 50% of stearin was reduced to 13% and 8%, respectively. Zhou et al.^15^ showed that stearin leads to growth inhibition in some microalgal species and acute mortality in some invertebrate species in the aquatic environment^15^. The last few years have seen an increased interest in petroleum/paraffin-based pesticides which have been applied for their insecticidal properties. These facts additionally point out that bee-wax adulterated with paraffin or stearin might severely deteriorate the honeybee physiology, as well. Thus, we already know that paraffin addition mostly leads to a deterioration of the mechanical properties of the combs, whereas stearin inclusions increase brood mortality^9^. We do not know, however, whether stearin and paraffin added to wax foundation would detract from the honeybee biochemical defense, particularly its all important component which is the antioxidant system^16^. Filling this gap in this knowledge is important, as the exposure of honeybees to numerous anthropogenic toxic compounds translates into increased production of free radicals and intensification of oxidative stress^17,18^. Consequently, we hypothesize that the contact of honeybees at their brood stage with bee-wax adulterated with paraffin or stearin may enhance the production of reactive oxygen species (ROS), which, apart from decreasing brood survival, could also weaken the biochemical defense of adult honeybee workers.

The main components of the antioxidative apian barrier are 3 enzymes: superoxide dismutase (SOD, transforms ROS into hydrogen peroxide and molecular oxygen), catalase (CAT, reduces hydrogen peroxide to molecular oxygen and water), glutathione peroxidase (GPx, reduction of glutathione to an oxidized form). The second line of defense is ensured by the enzyme glutathione which complements the action of the above (participates in changes that detoxify oxygen). All these enzymes work to capture free radicals and transform them into their non-toxic, less reactive forms^19^. Total antioxidant capacity (TAC) is, in turn, an indicator of the total level of the organism antioxidant capacity. Honeybees also have vitellogenin (Vg), which is a zinc-binding glycolipoprotein. Among many other pivotal functions, it not only positively influences the humoral and cell-based immunity^20^, but also fosters honeybee worker resistance to oxidative damage. By improving honeybee tolerance to oxidative stress, Vg also extends the honeybee life span^21,22^. Therefore, to corroborate our hypothesis, it is necessary to assess whether and how stearin and paraffin affect all these antioxidative parameters, including Vg. For this reason, we included honeybee workers aged 1, 7 and 14 days in our study.

Honeybee brood seemed to be relatively well protected against the harmful external environment within the honeybee colony, at least before we introduced the bee-wax adulterated with paraffin or stearin. On the other hand, nowadays, not only larvae but also adult bees like workers have to face many harmful anthropogenic external factors. That is why the novel and important requirement arises to study the effectiveness of the antioxidative systems of adult worker bees in a situation where the larvae from which they emerged were exposed to adulterated beeswax. Paleolog et al. (2011)^23^ and Burzyński et al. (2013)^24^ showed that feeding honeybee larvae with bioactive compounds causes changes in survival and biochemical characteristics, including global DNA methylation (DNA-met), in adult workers. Therefore, we want to specify our hypothesis that assumes that the addition of paraffin or stearin to the wax foundation could weaken the antioxidant barriers of the worker bees by affecting the larvae they emerged from, even though the workers were not subsequently exposed to these compounds. Confirming this hypothesis^1^ will expand the knowledge of the physiopathology of bees developing in the preimaginary period on adulterated beeswax, Blande (2021)^3^ will develop the awareness of how harmful the adulteration of wax foundations with paraffin and stearin can be, and^4^ may help to develop the proper honeybee food supplementation procedures when the insects are exposed to these harmful compounds.

The aim of our study was to determine the effect of both paraffin or stearin, when added to the wax foundation at concentrations of 10%, 30%, and 50%, on the activities of antioxidant enzymes (SOD, CAT, GPx, and GST), as well as on the levels of TAC, Vg, and DNA-met in adult honeybees that emerged from larvae reared on combs built on these foundations^9,25^.

## Materials and methods

### Obtaining workers and collecting material for analysis

The experiment was carried out at the apiary (51º13’36’’N, 022º38’5’’E) and laboratory of the University of Life Sciences in Lublin. The experiment was conducted in nucleus colonies with a small frames (210 mm × 170 mm). This made it possible to rear bees in combs built on a foundation adulterated with paraffin. With a larger surface area, such combs are significantly deformed Chęć et al. (2021)^9^, which prevents effective brood rearing. In order to obtain combs built up on adulterated wax and pure wax foundation, 9 strong colonies were selected. Each occupied four brood chambers with six combs in each chamber. In the first three colonies, three frames with pure wax foundation were placed in each (C – control, own pure wax). In the next three colonies, three frames with foundations adulterated with paraffin were placed, with a different level of adulteration in each colony – 10%, 30% and 50% of paraffin (PA10%, PA30%, PA50%), respectively. In the next three colonies, four frames with foundations adulterated with stearin were placed, with a different level of adulteration in each colony – 10%, 30% or 50% of stearin (ST10%, ST30%, ST50%), respectively. The use of a higher number of frames with foundations adulterated with stearin was caused by the need to obtain an appropriate number of bees. Brood survival is significantly reduced in combs built on a foundation adulterated with stearin Chęć et al. (2021)^9^. These experimental combs were protected against queen egg-laying with queen excluders at the time of building them. Then, 3 combs from each of these 3 types based on paraffin-adulterated and pure wax foundations (PA10%, PA30%, PA50%, and C) and 4 combs from each of these 3 types based on stearin-adulterated foundations (ST10%, ST30%, ST50%) were placed individually within the queen-excluder comb-cage and introduced separately into the experimental colonies. All the 21 colonies (7 group x 3 colonies) obtained in this way were headed by egg-laying queens and were of the similar strength and structure. Subsequently, the bee queens in each of the experimental colonies were caged within the comb-cage containing the experimental comb for 48 h for egg laying. Twenty days after laying the eggs, the capped brood combs were removed from the comb-cages, transferred to the laboratory and incubated separately to obtain one-day-old workers. Hemolymph was individually collected from 30 of these one-day-old workers from each of the 3 experimental combs, in the groups ST10%, ST30%, ST50%, PA10%, PA30%, PA50%, and C (30 workers x 3 combs x 7 groups = 630 workers), respectively. Additionally, 300 one-day-old workers were marked with a different color separately within ST10%, ST30%, ST50%, PA10%, PA30%, PA50%, and C (POSCA PC-3 M marker; 2100 workers) and introduced all together into one strong colony with combs built up from the own-produced, pure wax. Subsequently, hemolymph was collected from 30 marked workers within each group, when the insects attained the age of 7 and 14 days, respectively. Finally, the data base was constituted by 3 age classes (1, 7 and 14 days) x 30 workers x 7 groups = 630 individual hemolymph samples.

### Laboratory analyses

#### Hemolymph collection

To obtain the fresh hemolymph, a glass capillary (20 µL; the ‘end to end’ type; without anticoagulant; Medlab Products, Raszyn, Poland) was individually inserted between the third and fourth tergite of a living worker, according to the Łoś and Strachecka (2018)^26^ method. The hemolymph volumes were separately measured in each capillary. Hemolymph from an individual bee was collected into one sterile Eppendorf tube containing 25 µL of ice-cooled 0.6% NaCl. The hemolymph solutions were immediately refrigerated at − 40 °C for further biochemical analyses.

#### Bee brain collection and global DNA methylation determination

After collecting hemolymph, the brains were dissected from each worker and refrigerated (− 40 °C). After thawing, DNA was individually extracted from each bee’s brain using the DNeasy Blood and Tissue Kit (Qiagen, Hilden, Germany) following the manufacturer’s instructions. DNA was stored at − 25 °C, and then used to determine global DNA methylation with the Imprint Methylated DNA Quantification Kit (MDQ1-96RXN-Sigma, Ronkonkoma, NY, USA). The manufacturers’ instructions were followed during DNA extraction, as well as in the calculation of the global DNA methylation percentage.

#### Biochemical analyses

The antioxidant activities were measured in hemolymph solutions with the following kits:

- **SOD**; determined using the commercial Sigma-Aldrich (19160) SOD Determination Kit;
- **GPx**; determined using a commercial colorimetric test Peroxidase Activity Assay Kit (MAK092-1KT) from Sigma Aldrich;
- **CAT**; determined using the Catalase Assay Kit (219265-1KIT) from Sigma-Aldrich;
- **GST**; determined using the Glutathione-S-Transferase Assay Kit (CS0410-1KT) from Sigma-Aldrich.

All the antioxidant enzyme activities were calculated per 1 mg of protein.

- **Vg** level was determined using a Honey Bee Vitellogenin (VG) ELISA Kit (MyBioSource, Warsaw, Poland; MBS109137). This commercially available ELISA kit measures the Vg content in body fluids, tissue homogenates, secretions, or honeybee feces. The sensitivity of the test is 5 ng/mL. The measuring range is 31.2 ng/mL–1000 ng/mL (1.56–50 ng).
- **TAC** level was determined using the Total Antioxidant Capacity Assay Kit (MAK187-1KT) from Sigma-Aldrich.

The producer instructions attached to the tests were used.

### Statistical analysis

The results were analyzed using Statistica software formulas, version 13.3 (2017) for Windows, StatSoft Inc., USA. The type of distribution of the data was analyzed with the Shapiro-Wilk test. The distribution of these data appeared to be abnormal, therefore Kruskal-Wallis test was used to estimate the significance of the effects of the wax adulteration levels (PA10%, PA30%, PA50%, ST10%, ST30%, ST50% and C) on each of the biochemical characteristics (SOD, CAT, GPx, GST, TAC, Vg and DNA-met) within each of the age classes (1, 7 d and 14 days), separately for stearin and paraffin. The Kruskal-Wallis test also assessed the significance of the effects of the age (1, 7 d and 14 days) class on each of the biochemical parameter levels (SOD, CAT, GPx, GST, TAC, Vg and DNA-met) within each of the adulteration levels (PA10%, PA30%, PA50%, ST10%, ST30%, ST50% and C), separately for stearin, paraffin and the control.

As the effects of paraffin or stearin appeared adulteration level for each age classes (1, 7 d and 14 days) for each biochemical parameter levels (SOD, CAT, GPx, GST, TAC, Vg and DNA-met) to be significant (Table 1.), the comparisons of PA10%, PA30%, and PA50%, as well comparisons of ST10%, ST30%, and ST50% with the proper values of C, within each of the age classes (1, 7 d and 14 days), for each of the biochemical parameters separately (PA10%, PA30%, PA50%, ST10%, ST30%, ST50% and C), were performed using the Mann-Whitney U test (non-normally distributed data). The same test was used to compare ST10% with PA10%, ST30% with PA30%, and ST50% with PA50% within every age class (1, 7 d and 14 days), for each of the biochemical characteristics separately (SOD, CAT, GPx, GST, TAC, Vg and DNA-met) and for comparison within each adulteration level (PA10%, PA30%, PA50%, ST10%, ST30%, ST50% and C) between the age classes (1, 7 d and 14 days) for each of the biochemical characteristics separately (SOD, CAT, GPx, GST, TAC, Vg and DNA-met).

## Results

Both the effects of the adulteration level and worker age were highly significant (Tables 1 and 2). The adulteration of bee-wax with paraffin, as well as with stearin, significantly suppressed the antioxidant system (all the parameters) in the worker bees’ hemolymph (Figs. 1 and 2). The higher the adulteration level, the stronger the suppression was. The suppression caused by stearin was substantially stronger than that exerted by paraffin. In the case of stearin, even 10% adulteration caused a markedly higher decrease in the activities/levels of the antioxidant parameters. Surprisingly, as the worker bees grew older, the suppression effect increased, while the antioxidant parameter values rose along with age in Group C, at the same time (Fig. 1).

**Fig. 1 The effect of wax foundation adulteration with paraffin or stearin on the activities of the antioxidant enzymes (mean ± SD; n = 30) within the three age classes of the worker bees. Explanations: SOD – superoxide dismutase; CAT – catalase; GST – glutathione S- transferase; GPx – glutathione peroxidase. The wax foundations were adulterated with 10%, 30%, and 50% of paraffin (groups PA10%, PA30%, and PA50%), respectively. The wax foundations were adulterated with 10%, 30%, and 50% of stearin (groups ST10%, ST30%, and ST50%), respectively. Pure wax foundations with no additives were used (the control group C). (*) – the difference between the control group and a given experimental group was statistically significant (p ≤ 0.01) when evaluated within each age class. The horizontal green lines indicate that the specific group means differ significantly (p ≤ 0.01) for a given adulteration level when the bees reared on the stearin-adulterated combs are compared with those reared on the combs with paraffin, separately within each age class. a, b, c – the differences between the age groups for the control group and the particular levels of paraffin and stearin adulteration are statistically significant at p ≤ 0.01. The vertical bars indicate standard deviation.**
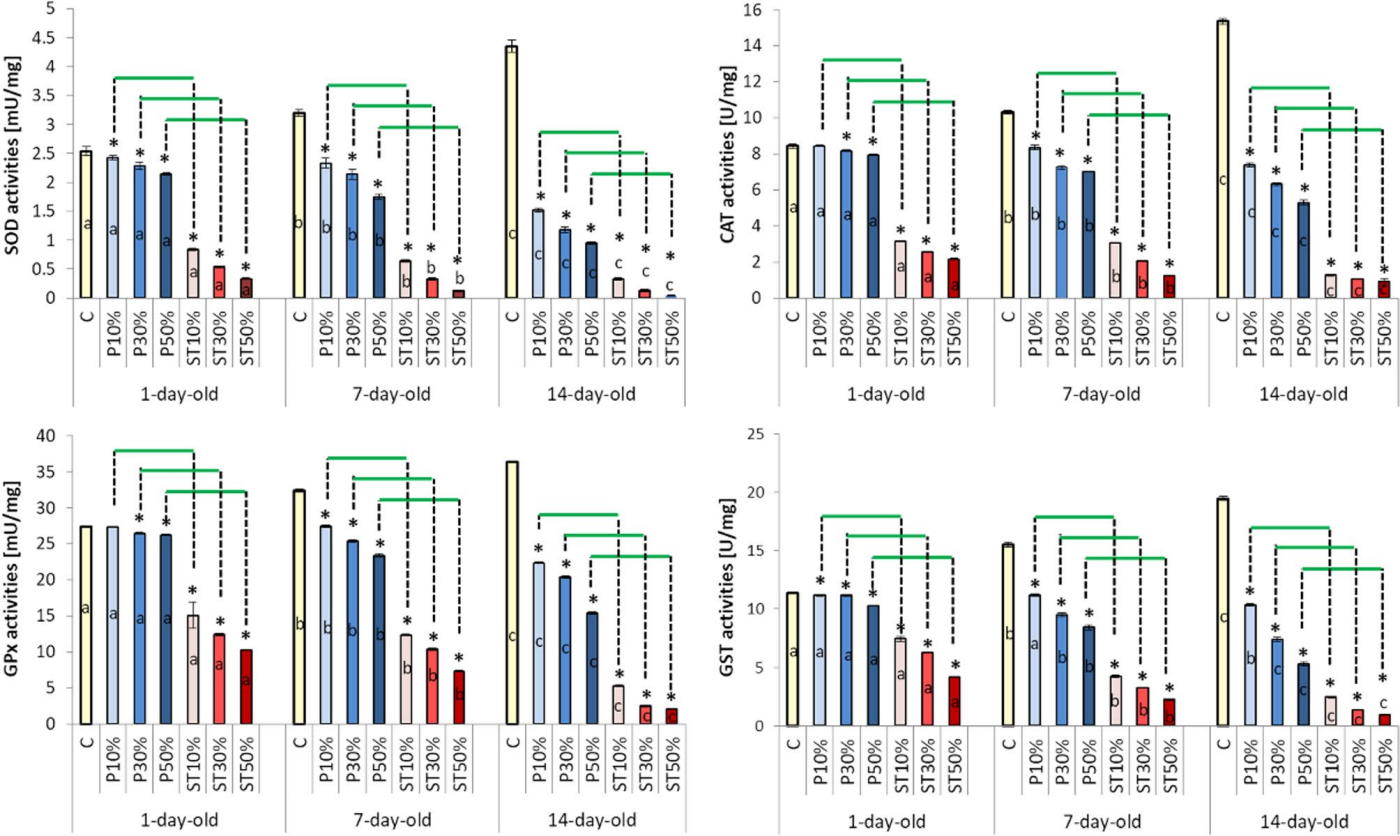
.

**Fig. 2 The effects (mean ± SD; n=30) of wax foundation adulteration with paraffin or stearin on the total antioxidant capacity (TAC) within the three age classes of the worker bees. Explanations: The wax foundations were adulterated with 10%, 30%, and 50% of paraffin (groups PA10%, PA30%, and PA50%), respectively. The wax foundations were adulterated with 10%, 30%, and 50% of stearin (groups ST10%, ST30%, and ST50%), respectively. Pure wax foundations with no additives were used (the control group C). (*) – the difference between the control group and a given experimental group was statistically significant (p ≤ 0.01) when evaluated within each age class. The horizontal green lines indicate that the specific group means differ significantly (p ≤ 0.01) at a given adulteration level when the bees reared on the stearin-adulterated combs are compared with those raised on the combs with paraffin, separately within each age class. a, b, c – the differences between the age groups for the control group and the particular levels of paraffin and stearin adulteration are statistically significant at p ≤ 0.01. The vertical bars indicate standard deviation.**
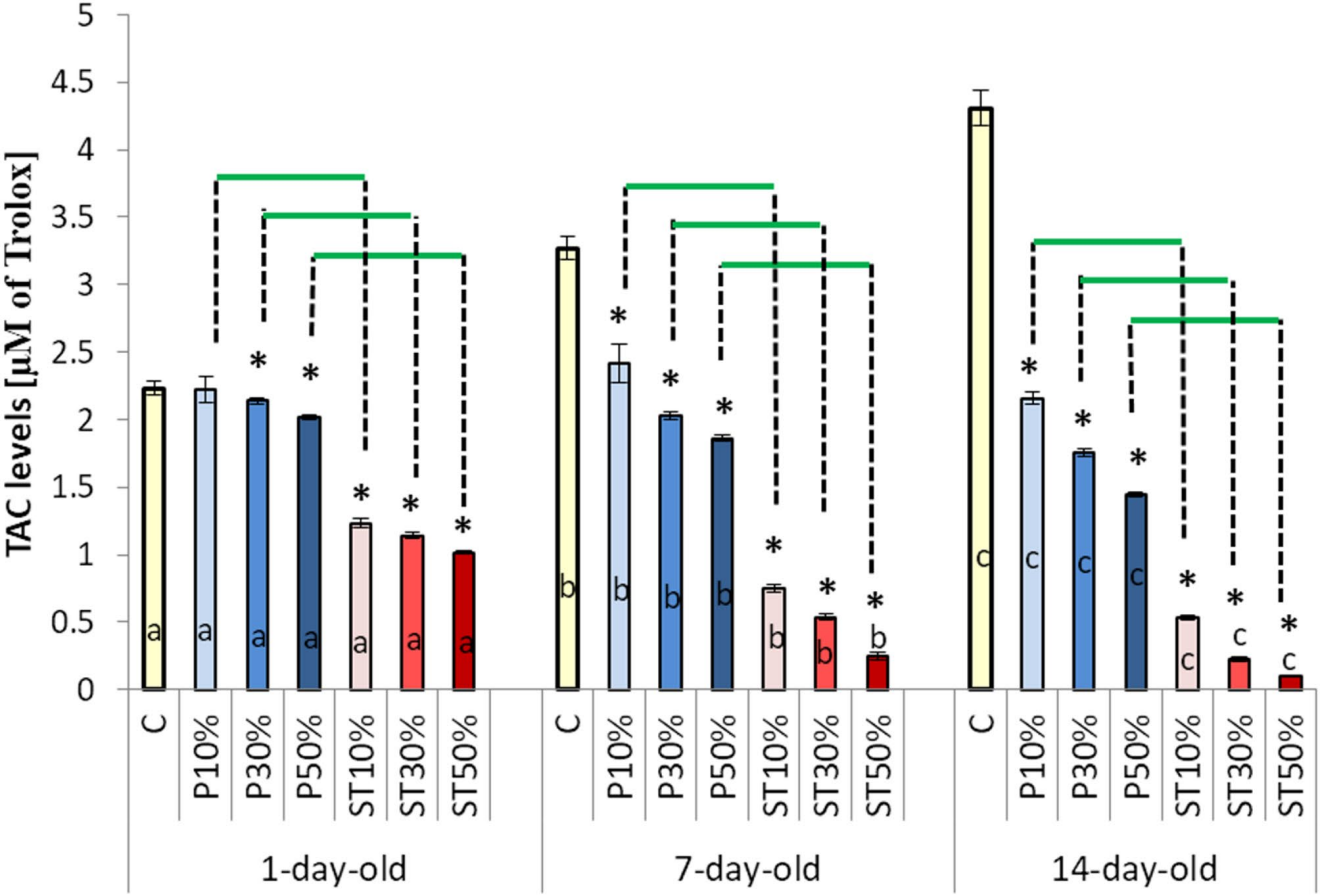
.

Vg titers decreased with age in all the groups, including Group C. However, paraffin, and particularly stearin, substantially increased this decline. The older the bees, the greater the decline was (Fig. 3).

**Fig. 3 The effects of wax foundation adulteration with paraffin or stearin on the vitellogenin (Vg) titers (mean ± SD; n = 30) within the three age classes of worker bees. Explanations: The wax foundations were adulterated with 10%, 30%, and 50% of paraffin (groups PA10%, PA30%, and PA50%), respectively. The wax foundations were adulterated with 10%, 30%, and 50% of stearin (groups ST10%, ST30%, and ST50%), respectively. Pure wax foundations with no additives were used (the control group C). (*) – the difference between the control group and a given experimental group was statistically significant (p ≤ 0.01) when evaluated within each age class. The horizontal green lines indicate that the specific group means differ significantly (p ≤ 0.01) for a given adulteration level when the bees reared on the stearin-adulterated combs are compared with those reared on the combs with paraffin, separately within each age class. a, b, c – the differences between the age groups for the control group and the particular levels of paraffin and stearin adulteration are statistically significant at p ≤ 0.01. The vertical bars indicate standard deviation.**
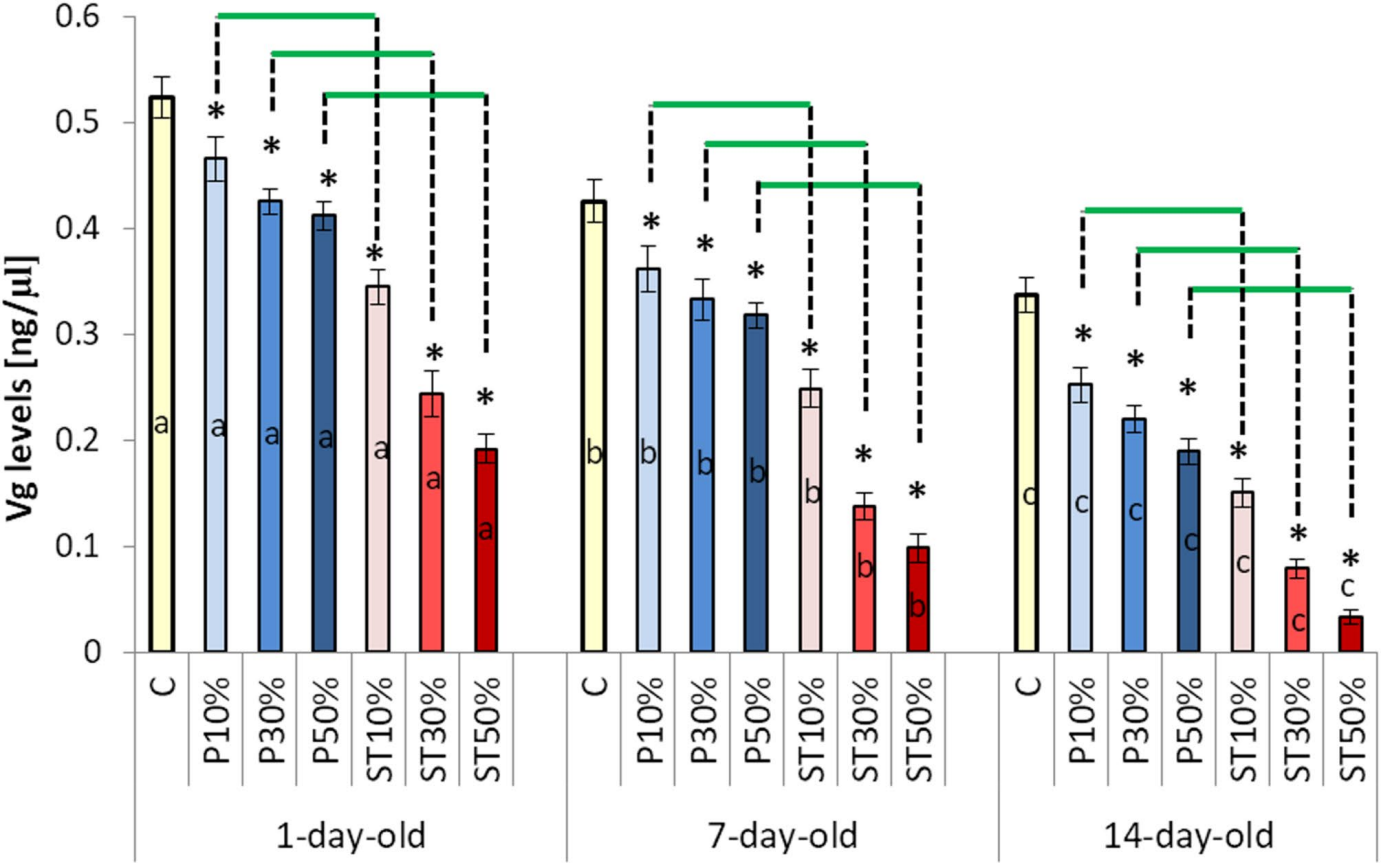
.

**Table 1:** The effects of the adulteration level in the particular age classes on the level of each of the biochemical parameters estimated separately in the worker bees emerged from the brood reared in combs built on wax foundations adulterated with paraffin or stearin.

Biochemical parameters	Effect of adulteration level					
	Paraffin			Stearin		
	worker age			worker age		
	1-day	7-day	14-day	1-day	7-day	14-day
SOD	H_3_ = 105.00 *p* ≤ 0.01	H_3_ = 109.42 *p* ≤ 0.01	H_3_ = 111.58 *p* ≤ 0.01	H_3_ = 111.61 *p* ≤ 0.01	H_3_ = 111.58 *p* ≤ 0.01	H_3_ = 111.57 *p* ≤ 0.01
CAT	H_3_ = 99.22 *p* ≤ 0.01	H_3_ = 111.48 *p* ≤ 0.01	H_3_ = 111.57 *p* ≤ 0.01	H_3_ = 111.59 *p* ≤ 0.01	H_3_ = 111.57 *p* ≤ 0.01	H_3_ = 111.58 *p* ≤ 0.01
GPx	H_3_ = 98.06 *p* ≤ 0.01	H_3_ = 111.60 *p* ≤ 0.01	H_3_ = 111.62 *p* ≤ 0.01	H_3_ = 107.29 *p* ≤ 0.01	H_3_ = 111.58 *p* ≤ 0.01	H_3_ = 111.49 *p* = 0.00
GST	H_3_ = 97.09 *p* ≤ 0.01	H_3_ = 111.59 *p* ≤ 0.01	H_3_ = 111.59 *p* ≤ 0.01	H_3_ = 111.57 *p* ≤ 0.01	H_3_ = 111.59 *p* ≤ 0.01	H_3_ = 111.60 *p* ≤ 0.01
Vg	H_3_ = 99.36 *p* ≤ 0.01	H_3_ = 93.91 *p* ≤ 0.01	H_3_ = 108.39 *p* ≤ 0.01	H_3_ = 110.96 *p* ≤ 0.01	H_3_ = 110.77 *p* ≤ 0.01	H_3_ = 109.70 *p* ≤ 0.01
TAC	H_3_ = 88.06 *p* ≤ 0.01	H_3_ = 111.60 *p* ≤ 0.01	H_3_ = 111.58 *p* ≤ 0.01	H_3_ = 110.17 *p* ≤ 0.01	H_3_ = 111.61 *p* ≤ 0.01	H_3_ = 111.59 *p* ≤ 0.01
DNA-met	H_3_ = 35.53 *p* ≤ 0.01	H_3_ = 36.61 *p* ≤ 0.01	H_3_ = 36.59 *p* ≤ 0.01	H_3_ = 36.60 *p* ≤ 0.01	H_3_ = 36.61 *p* ≤ 0.01	H_3_ = 36.59 *p* ≤ 0.01

Explanations: H – statistical value for the Kruskal–Wallis test; _3_ – number of degrees of freedom; p - probability value. Superoxide dismutase (SOD); catalase (CAT), glutathione peroxidase (GPx); total antioxidants capacity (TAC); vitellogenin (Vg); global DNA methylation (DNA-met). The wax foundations were adulterated with 10%, 30%, and 50% of paraffin (PA10%, PA30% and PA50%), respectively. The wax foundations were adulterated with 10%, 30%, and 50% of stearin (ST10%, ST30%, and ST50%), respectively. Pure wax foundations with no additives were used (C).

**Table 2:** The effects of the age class at the particular adulteration levels on each of the biochemical parameters estimated separately in the worker bees emerged from the brood reared in combs built on wax foundations adulterated with paraffin or stearin and in the control group.

Biochemical parameters	Effect of age						
	Paraffin			Stearin			Control pure wax – no additives
	PA10%	PA30%	PA50%	ST10%	ST30%	ST50%	
SOD	H_2_ = 68.71 *p* ≤ 0.01	H_2_ = 72.38 *p* ≤ 0.01	H_2_ = 79.16 *p* ≤ 0.01	H_2_ = 79.17 *p* ≤ 0.01	H_2_ = 79.13 *p* ≤ 0.01	H_2_ = 79.15 *p* ≤ 0.01	H_2_ = 79.12 *p* ≤ 0.01
CAT	H_2_ = 66.60 *p* ≤ 0.01	H_2_ = 79.13 *p* ≤ 0.01	H_2_ = 79.13 *p* ≤ 0.01	H_2_ = 79.15 *p* ≤ 0.01	H_2_ = 79.13 *p* ≤ 0.01	H_2_ = 79.14 *p* ≤ 0.01	H_2_ = 79.13 *p* ≤ 0.01
GPx	H_2_ = 62.29 *p* ≤ 0.01	H_2_ = 79.15 *p* ≤ 0.01	H_2_ = 79.18 *p* ≤ 0.01	H_2_ = 75.39 *p* ≤ 0.01	H_2_ = 79.16 *p* ≤ 0.01	H_2_ = 79.12 *p* ≤ 0.01	H_2_ = 79.15 *p* ≤ 0.01
GST	H_2_ = 59.77 *p* ≤ 0.01	H_2_ = 79.14 *p* ≤ 0.01	H_2_ = 79.13 *p* ≤ 0.01	H_2_ = 79.15 *p* ≤ 0.01	H_2_ = 79.13 *p* ≤ 0.01	H_2_ = 79.14 *p* ≤ 0.01	H_2_ = 79.17 *p* ≤ 0.01
Vg	H_2_ = 79.19 *p* ≤ 0.01	H_2_ = 78.71 *p* ≤ 0.01	H_2_ = 79.14 *p* ≤ 0.01	H_2_ = 79.16 *p* ≤ 0.01	H_2_ = 79.16 *p* ≤ 0.01	H_2_ = 79.14 *p* ≤ 0.01	H_2_ = 79.14 *p* ≤ 0.01
TAC	H_2_ = 51.69 *p* ≤ 0.01	H_2_ = 78.71 *p* ≤ 0.01	H_2_ = 79.17 *p* ≤ 0.01	H_2_ = 79.17 *p* ≤ 0.01	H_2_ = 79.16 *p* ≤ 0.01	H_2_ = 79.15 *p* ≤ 0.01	H_2_ = 79.15 *p* ≤ 0.01
DNA-met	H_2_ = 25.86 *p* ≤ 0.01	H_2_ = 25.82 *p* ≤ 0.01	H_2_ = 25.82 *p* ≤ 0.01	H_2_ = 25.83 *p* ≤ 0.01	H_2_ = 25.82 *p* ≤ 0.01	H_2_ = 25.84 *p* ≤ 0.01	H_2_ = 25.82 *p* ≤ 0.01

Explanations: H – statistical value for the Kruskal–Wallis test; _2_ – number of degrees of freedom; p - probability value. Superoxide dismutase (SOD); catalase (CAT), glutathione peroxidase (GPx); total antioxidants capacity (TAC), vitellogenin (Vg), global DNA methylation (DNA-met). The wax foundations were adulterated with 10%, 30%, and 50% of paraffin (PA10%, PA30% and PA50%), respectively. The wax foundations were adulterated with 10%, 30%, and 50% of stearin (ST10%, ST30%, and ST50%), respectively. Pure wax foundations with no additives were used (C).

**Fig. 4 The effects of wax foundation adulteration with paraffin or stearin on the global DNA methylation level (mean ± SD; n = 30) within the three age classes of worker bees Explanations: The wax foundations were adulterated with 10%, 30%, and 50% of paraffin (groups PA10%, PA30%, and PA50%), respectively. The wax foundations were adulterated with 10%, 30%, and 50% of stearin (groups ST10%, ST30%, and ST50%), respectively. Pure wax foundations with no additives were used (the control group C). (*) – the difference between the control group and a given experimental group was statistically significant (p ≤ 0.01) when evaluated within each age class. The horizontal green lines indicate that the specific group means differ significantly (p ≤ 0.01) for a given adulteration level when the bees reared on the stearin-adulterated combs are compared with those raised on the combs with paraffin, separately within each age class. a, b, c – the differences between the age groups for the control group and the particular levels of adulteration with paraffin and stearin are statistically significant at p ≤ 0.01. The vertical bars indicate standard deviation.**
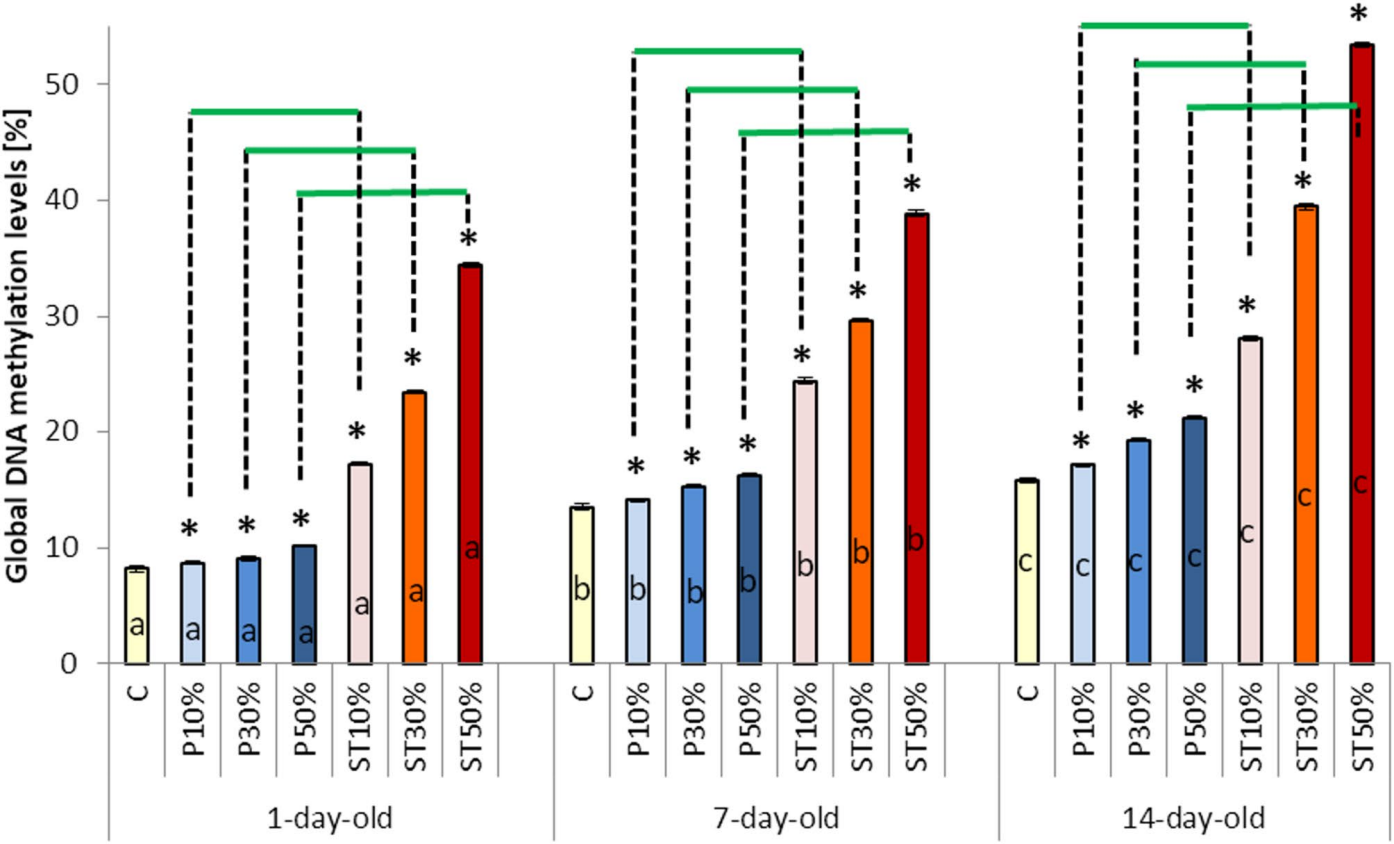
.

DNA-met increased with age in Group C (Fig. 4). The same tendency was observed in the PA10%, PA30% PA50%, ST10%, ST30%, and ST50% groups. However, the increases in these groups were markedly higher than in Group C. Hence, it can be gathered that paraffin/stearin accelerated DNA-met. The older the bees, the greater the acceleration of DNA methylation, and the higher the level of DNA-met. Again, this increase was particularly evident when the wax was adulterated with stearin, which substantially accelerated the methylation.

What is worth emphasizing is the fact that the values of standard deviation were relatively low for all the parameters in comparison with the respective means. This confirms that in each group, i.e. PA10%, PA30%, PA50%, ST10%, ST30%, ST50%, and C, all the characteristics were affected evidently, clearly and the effects were not markedly influenced by undefined, unknown factors, including errors of the applied methods (e.g. sampling, storage and processing of hemolymph, analytical procedures, etc.).

## Discussion

### Antioxidative enzymes and total antioxidant capacity

Paleolog et al. (2021)^27^ and Skowronek et al. (2022, 2023)^28,29^ have shown that the activity of the antioxidant system increases with the age of worker bees. Smirle and Winston (1998)^30^Corona et al. (2005)^31^Carey (2014)^32^ suggested that such an increase may reflect the age-related biochemical adaptation of the worker to the subsequent tasks associated with the rising risk of exposition to harmful environmental factors – particularly to increasing oxidative stress. This could be particularly necessary for the contemporary honeybees dwelling in the anthropogenically polluted ecosystems^3,33,34^. In this study, we have also observed the same age-dependent rise of the antioxidative abilities in the worker bees in our group C that could be considered typical^22^. Quite conversely, the workers that had emerged from the combs built on the stearin- or paraffin-adulterated wax foundations, had strongly suppressed antioxidative systems. This suppression became stronger as the workers aged. Secondly, it is striking that all the parameters of the antioxidative system were suppressed in the bees exposed to paraffin/stearin already at the brood stage (Figs. 1, 2 and 3; Table 1). It can be assumed that the first chemical reactions between stearin or paraffin in the wax and the compounds/components of the honeybee organism take place already in the egg and then in the larvae and pupae until the workers emerge (1-day-old workers), with low antioxidant activities in comparison with Group C. Stearin and paraffin can interact with the egg chorion and disrupt the chitin synthesis pathway involved in the formation of the chorion which is important for embryo respiration and for preventing dehydration^35^. A poorly formed chorion in a honeybee egg is the cause the death of an embryo or its developmental defects (e.g. the disruption of the morphology of the larva) before the hatching process begins^36^. When the larvae hatch from honeybee eggs, a fluid is released that enzymatically dissolves the chorion^37^. Stearin and paraffin may react with the enzymes of this fluid, resulting in errors, which could then lead to the suppression of the antioxidative system in the adult worker bees. It can therefore be concluded that the contact of the preimaginal stages with wax adulterated with stearin and paraffin resulted in the destabilization of the antioxidant barrier in the adult workers.

Have Lee et al. (2017)^38^ and Paleolog et al. (2021)^27^ have shown that even though the total antioxidant capacity was decreased due to the exposition to a harmful xenobiotic (e.g. pesticides), some of the antioxidant enzymes were suppressed, while others were not. Hence, the activity of a specific antioxidative enzyme does not have to correlate with the total antioxidant capacity. In our experiment, however, all the antioxidative parameters were suppressed without any exception. Therefore, the suppression must have been serious. Moreover, the age-related suppression of the antioxidant system in our worker bees exposed to paraffin/stearin at the brood stage turns out to be really significant when evaluated in relation to Group C, in which the antioxidative abilities increased with age. All these findings may be considered disturbing for honeybee breeders and ecologists.

Summing up, paraffin or stearin added to the wax foundation unexpectedly strongly suppressed the antioxidant system in our worker bees emerged from larvae reared on such combs. This is the first important new finding, which not only expands our knowledge, but is also a warning to wax foundation producers and beekeepers. Consequently, we suggest to add stearin/paraffin to the list of anthropogenic factors, which could be responsible for honeybee colony depopulation, not only because the compounds increase brood mortality^9,10^ but also by weaken the biochemical defense of adult bees exposed to many harmful xenobiotics in the contemporary ecosystems^3,17^. This corresponds with the suggestions of Dong et al. (2022)^18^ that decreased activity of the antioxidant defense disturbs the regenerative abilities of the bee organism. As stearin appeared to be much more harmful than paraffin it should be considered in the first place. We also want to mention here that the free radical theory of aging is applicable to honeybees^27,39^ while senescence and resistance to the oxidative stress are interconnected in social insects^22^. Thus, particularly stearin may decrease the worker bees’ longevity.

### Vitellogenin and DNA global methylation

ROS-scavenging antioxidant enzymes such as CAT, GPx and SOD constitute the first line of the antioxidative defense in honeybees when they are exposed to harmful xenobiotics^40^. These enzymes are supported by GST which acts as the second line of defense. The activities of these four enzymes are often suppressed by xenobiotics, even though they should increase with age^41,42^. Moreover, various physiological processes are regulated by Vg, which is functionally related to hormones (e.g. juvenile hormone) and hemocytes, and at the same time supports the first and second lines of antioxidant defense (by shielding cells against oxidative damage and improving cell tolerance to oxidative stress)^19,20,42,43^. Its antioxidant abilities influence of lifespan of queen and worker bees^20,44–46^. Moreover, Vg might help honeybees to maintain the high efficiency of their antioxidant defense when its first line is suppressed – as may have been the case with pesticides, e.g. imidacloprid^21,39^. In this study we revealed for the first time that rearing the brood on the wax foundation adulterated with paraffin, and particularly stearin, significantly decreased the Vg titers in the worker bees emerged from such brood. This is the second important finding of our study, which expands the knowledge about potential suppressors of Vg. Therefore, the first and the second line of the antioxidant defense could be weakened by adulterated wax, particularly by stearin. It is natural for the Vg titers to decrease as workers age^47–50^. However, a subthreshold, an unnatural decrease in the level of Vg in the hemolymph of workers of all ages (compared to healthy bees, with controls) may not only cause a dysfunction in the resistance to oxidative stress but also contribute to a dysfunction in the physiological development and immune responses, such as increased susceptibility to parasites and pathogens. Amdam et al. (2009)^46^ showed that Vg down-regulation results in an elevated JH level and an increase in the bee’s responsiveness to sucrose. It can therefore be concluded that the consequence of adulterating foundations is the dysregulation of the metabolism of the apian organism and most likely a shortened lifespan. Or perhaps, Antonio et al. (2008)^49^ has shown, as a result of vitellogenin depletion (which is particularly visible in the groups of experimental bees, Fig. 3), bees start foraging flights more quickly.

Summing up, decreased Vg titers in bees kept at the brood stage on wax combs adulterated with paraffin, and above all with stearin, may cause the insects to be more sensitive to biotic and abiotic anthropogenic harmful factors through subdued not only first but also second lines of apian antioxidative defense which depends on Vg and other antioxidants. Furthermore, this decrease may also result in a wider dysregulation of the entire honeybee worker physiology. It is not good information for beekeepers exposed to adulterated wax foundations.

DNA-met level is often altered by environmental changes. It is the “epigenetic tool” used by insect organisms to effectively respond to environmental pressure^51,52^. For instance DNA-met was significantly accelerated by the imidacloprid pesticide and other harmful xenobiotics^33^. The DNA-met levels increase with age in honeybees^24,52^. On the other hand, RG108 (N-Phthalyl-L-tryptophan) induces genome demethylation, increasing honeybee lifespan^53^. Curcumin, while decreasing DNA-met, also expanded honeybee longevity^42^. In our study, the level of DNA-met was, expectedly, slowly increasing with age in the worker bees in Group C. The addition of paraffin to the combs, from which these bees emerged, increased the DNA-met levels. However, when stearin was added, the DNA-met levels rose really significantly. This suggests that the epigenetic response to stearin was very pronounced. This feature of stearin had not been previously known. The older the bees were, the stronger the response was. Importantly, our bees were exposed to stearin at the brood stage and did not remain in contact with stearin after their emergence. The increase in gene expression (including chromatin remodeling, the TCA cycle and the glutathione system) and the decrease in DNA-met levels, accompanied with extended apian longevity, were observed in adult honeybee workers when the “epigenetic switches” phenylbutyrate or phenylacetylglutaminate were added to the larval diet^50^. Hence, stearin could be considered as a substantial “epigenetic switch” that might accelerate worker bees’ ageing. Consequently, the suppression of the antioxidant defense in workers emerged from the brood reared on combs built on the stearin- or paraffin-adulterated wax foundations, may have the epigenetic background. Paraffin or stearin had long-term effects, as the levels of all the biochemical parameters were lower in the older bees than in the younger ones, which confirms the above suggestion. This is the third new significant information that expands our knowledge of the mechanisms of harmful influence of stearin on the physiology of honeybee workers.

### Additional considerations

Our bees had to emerge from the only larvae, which had survived the exposure to stearin/paraffin and many larvae were dying when exposed to those compounds^9^. Thus, our bees originated from the most resistant larvae but at the same time might have had physiological defects. Many other external factors/stressors that honeybee pre-imaginal stages come into contact with may also affect the physiology of the adult bees that emerge from them^42,54,55^. Dziechciarz et al. (2023)^56^ showed that the activities of antioxidants in the hemolymph of adult workers are influenced even by the size of the cell in which they have developed as brood. The mechanism of stearin action may involve two stages. First, stearic acid reacts with chitin, leading to chemical modifications of its functional groups (esterification of 2 hydroxyl groups and deacetylation of the N-acetylamino group) and a loosening of the cuticle structure. Such damaged cuticle does not protect against water loss, mechanical damage and the entry of pathogens into the bee’s body^57^. Then, stearic acid penetrates the fat body and hemolymph of the brood and most likely inactivates the antioxidants. Moreover, Smutin et al. (2022)^58^ confirmed that the presence of harmful substances in a bee colony changes the microbiome of the bees and the hive itself. Paraffin can be a source of radicals, in particular hydroxyl radicals (ROS), formed in the hydroxylation process (e.g. during photolysis)^59^. Additionally, these compounds can take part in the radical halogenation reaction which can result in the formation of for example chloromethane or hydrogen chloride. Moreover, Ibrahim et al. (2020, 2021)^60,61^ showed that paraffin can be a nutrient for bacteria. Presumably, even if these bacteria are not pathogenic to bees, the metabolites they secrete may act as antioxidant inhibitors. This may result in the overproduction of free radicals, the neutralization of which depends on a well-functioning antioxidant system. Although honeybee adult and larval stages are effectively microbiologically decoupled, and the core adult microbiome is remarkably stable against early developmental perturbations, changes in the larval microbiome lead to morphological and physiological errors in the larvae^62^. Such errors will also be carried over to adult insects^63^ and, in our opinion, manifest themselves in a drastic decrease in antioxidant activities and levels of TAC and DNA-met in adult bees that developed in the preimaginal period on adulterated wax.

There is one serious problem with the combs adulterated with paraffin or stearin. Such combs are then used to produce new wax foundations which are used in turn to produce the next combs. Disposal or non-beekeeping use of them could be problematic due to the scarcity of wax on the market. Thus, it is very difficult to remove paraffin/stearin from honeybee colonies because it is in constant circulation. What can we do about it here and now? Studies of Strachecka et al. (2014a, 2014b, 2015)^41,42^ and Skowronek and Strachecka (2023)^28^ showed that curcumin, coenzyme Q10, caffeine, and cannabidiol (CBD) increase SOD, CAT, GPx and GST activities and the TAC levels causing a significant extension of workers’ lifespans, from 1 even up to 3 weeks^64^. Hence, the supplementation of bee food may alleviate the harmful effects of stearin/paraffin on honeybees.

The practice of honeybee wax adulteration with paraffin and stearin may also have other negative consequences, among others for the human health, as such wax is used as a food additive (E901)^65^. Moreover, it constitutes the basis of the cells in the combs in which honey and other bee products are stored (compare to Wilmart)^8^ Paraffin produced from the fraction of crude oil and refined depending on its intended use turned out to be toxic and carcinogenic not only in contact with the respiratory system but also in direct contact with the digestive system^8^. The addition of paraffin to the diet of rats, caused an enlargement of their livers and contributed to the development of histopathological changes^66^. Nunes et al. (2020) showed that large paraffin particles reduced GPx activity in mussels. Research on paraffin was focused on its negative impact on the respiratory system of mammals during paraffin combustion (the production of benzene and toluene)^68^. Therefore, the problem of wax adulteration should be also addressed by institutions responsible for food quality monitoring.

### Recommendations for further studies

Our results constitute a preliminary report which points out how harmful comb adulteration with paraffin/stearin may be to the antioxidative barriers of adult worker bees reared in such combs at the pre-imaginal stages. The impact was so evident and unexpectedly strong that it urgently calls for further studies in this area. Consequently, additional investigations aimed at developing procedures for routine quality evaluation and approval of manufactured wax substrates should be performed, as well. Our suggestion that stearin could be considered as an active “epigenetic switch” should also be confirmed. Could stearin reduce the honeybee life-span or interfere with the age-dependant division of tasks among worker bees by accelerating DNA-met and suppressing Vg? This is the question that should be answered, as well. Another task for new research we suggest is investigating whether the suppression of the honeybee antioxidative barrier and Vg could be compensated by supplementing the bee food with active biostimulators.

## Conclusions

This study has revealed for the first time that paraffin and stearin unexpectedly strongly suppressed the antioxidant system, including Vg, in honeybee workers emerged from larvae reared on combs adulterated with these compounds. Stearin appeared to be much more harmful and may cause unexpectedly serious metabolic disorders, including the acceleration of global DNA methylation. This is the important new information which not only expands our knowledge but also is a warning to wax foundation producers and beekeepers. Therefore, there is an urgent need to introduce proper procedures and regulations for the routine quality evaluation and approval of the manufactured wax.

Scientists and beekeepers should seriously reconsider rearing honeybee brood on wax foundations adulterated with paraffin/stearin as they can make worker bees more sensitive to unfavorable biotic and abiotic environmental factors by diminishing their antioxidative abilities.

We have pointed out for the first time that the suppression of the honeybee antioxidant barriers, particularly with stearin, may have an epigenetic background. This should also be taken into consideration as honeybees are a good model for epigenetic research.

Since the elimination of the adulterated bee-wax could be difficult, the supplementation of bee food with biostimulators that increase apian antioxidative abilities may be viable.

### Implications

The shortage of beeswax on the market stimulates the practice of adding adulterants to it, including paraffin and stearin. Until now, the focus has been on the physicochemical properties of adulterated wax and its impact on the development of larvae. We have proven that the consequences of using such wax in beekeeping are far-reaching and it can affect the decrease in the bees’ immunity by destabilizing the antioxidant system and epigenetic changes. As a result, colonies will be economically/productively inefficient for the beekeeper. This new information is also a warning to manufacturers of wax foundations. There is an urgent need to introduce procedures and regulations for the routine quality assessment and approval of manufactured wax.

## Data Availability

Raw data is available from the corresponding author. Anyone can access the raw data after sending a query. Moreover, the raw data is stored in the repository RepOD: https://doi.org/10.18150/JMWVRI.
